# A Digital Twin-Driven Dual-Stage Adversarial Transfer Learning Method for Lamb Wave-Based Structural Damage Localization Under Limited Sensing Data

**DOI:** 10.3390/s26051479

**Published:** 2026-02-26

**Authors:** Yuan Huang, Jiajia Yan, Qijian Liu

**Affiliations:** School of Aerospace Engineering, Xiamen University, Xiamen 361102, China; huangyuan1@stu.xmu.edu.cn (Y.H.); yanjiajia@xmu.edu.cn (J.Y.)

**Keywords:** limited sensing data, digital twin, dual-stage adversarial and transfer learning, damage localization, multi-objective optimization

## Abstract

Structural health monitoring (SHM) based on Lamb waves relies on sensors to acquire structural response signals. However, sensor data acquisition is severely constrained under complex damage conditions. Digital twins (DTs) can enhance damage monitoring capabilities in Lamb wave SHM by integrating simulation and experimental sensor data. Nevertheless, performance remains limited by discrepancies in signal distribution between digital and physical domains, as well as cross-domain optimization conflicts. This study proposes a digital twin-driven dual-stage adversarial and transfer learning method with multi-objective optimization (DT-DSATMO) for Lamb wave-based structural damage localization under limited sensing conditions. Firstly, a strategy for hierarchical feature enhancement and conditional generation incorporating physical prior knowledge is introduced to construct distribution-consistent feature representations in the digital domain. Secondly, it achieves adaptive alignment between the two domains via a lightweight domain adversarial transfer network, improving cross-domain feature transferability. Furthermore, a Pareto frontier-based multi-objective optimization strategy is employed to balance damage localization accuracy, cross-domain robustness, and feature consistency. The proposed method is experimentally validated on a representative aircraft wing-box panel equipped with four lead zirconate titanate (PZT) sensors. The case study results show that it substantially enhances damage localization accuracy and cross-domain generalization under limited sensing data.

## 1. Introduction

Large-scale engineering structures often exhibit nonlinear responses under complex environmental conditions and multi-source loads [1]. Progressive damage is difficult to observe directly, posing challenges for structural condition assessment and failure prediction [2,3]. Therefore, SHM has emerged as a key technology for achieving structural condition awareness, damage identification, and performance evaluation [4,5].

Current research methodologies in SHM can broadly be categorized as physics-driven and data-driven approaches. The former relies on analytical, semi-analytical, or finite element models (FEMs) to describe structural dynamics and wave propagation mechanisms, combined with imaging or delay sum algorithms for damage detection [6,7]. This approach offers strong physical interpretability, but incurs high modeling costs and computational overhead for complex structures or multiple damages. In contrast, data-driven techniques utilize machine learning to identify damage-related features from sensor signals. However, their reliance on large labeled datasets hinder generalization, particularly in environments where sensor data is scarce [8,9]. Recently, unsupervised machine learning techniques have been explored to mitigate the reliance on large labeled datasets. For instance, hierarchical clustering combined with the electromechanical impedance method [10] and generative adversarial networks integrated with acoustic emission sensing [11] have shown potential in small-sample scenarios. Nevertheless, these approaches often lack sufficient discriminative power and do not incorporate domain-specific physics knowledge, limiting their ability to capture subtle, damage-sensitive characteristics and hindering cross-domain generalization.

These limitations have motivated growing interest in hybrid frameworks that combine physical modeling with data-driven learning. DTs exemplify such integration, linking physical domain with sensor data and intelligent algorithms to enable damage diagnosis [12]. The core of DT-driven SHM lies in establishing mappings between digital and physical domains, enabling digital models to dynamically reflect a structure’s actual state [13,14]. Previous research has advanced this technique using low-fidelity physical models integrated with physics-informed neural networks [15] and discrete models combined with machine learning classifiers, achieving predictive accuracy [16]. Compared to traditional single-model approaches, DTs emphasize cross-domain consistent modeling and robust interaction, offering unique advantages for Lamb wave-based SHM [17]. Nonetheless, stable cross-domain mapping of damage features under complex wave propagation remains challenging.

Existing methods attempt to mitigate the discrepancies between the two domains through various model-adaptation strategies, including parameter calibration, multi-fidelity modeling, and retraining with limited experimental data. For instance, multi-fidelity modeling combined with deep generative models [18] or Bayesian calibration [19] has been employed to narrow the gap between digital and physical signals. However, these approaches mainly rely on costly labeled sensing data and treat cross-domain mapping as a posterior correction. They are sensitive to initialization and noise, underutilize prior knowledge in the digital domain, and often restrict cross-domain interactions to static parameter adjustments.

Nevertheless, even when an interaction between the two domains has been established, the optimal methods for constraining and optimizing such cross-domain sensor data interactions during damage localization processes remain underexplored. DT-driven damage localization is inherently a cross-domain problem, unlike conventional single-domain regression or classification. Existing approaches often rely on minimizing a single loss, assuming similar feature distributions between simulated and experimental signals, which rarely holds in practice [20,21,22]. Transfer learning and domain adaptation have been applied to reduce cross-domain discrepancies. For example, Zhao et al. and Mohammad et al. transferred discriminative knowledge from source-domain samples to the target domain to enhance domain invariance and discriminability [23,24]. However, overemphasis on distribution alignment can undermine feature discriminability. Without explicit constraints on cross-domain higher-order statistical discrepancies, feature drift may still accumulate in the latent space, exacerbating model performance instability [25]. For damage localization driven by digital twins, models must simultaneously balance localization accuracy, cross-domain robustness, and feature consistency across domains. These objectives are often mutually constraining, making unified optimization via a single loss function challenging. Consequently, developing dynamic multi-objective optimization strategies to manage these trade-offs remains a critical issue requiring resolution.

Although progress has been made, existing digital twin-driven damage localization approaches are still hindered by several critical challenges:

(1) Simplified modeling approaches that rely solely on point excitation and displacement output fail to capture the electromechanical coupling excitation and sensing mechanism of piezoelectric sensors. This results in significant deviations between simulated signals and experimental signals in terms of amplitude, excitation mode, and guided wave propagation.

(2) Cross-domain feature alignment frequently relies upon fine-tuning pretrained models. When sensor data is scarce, this process becomes highly susceptible to random initialization, optimization strategies, and sample noise, consequently leading to unstable model convergence, catastrophic forgetting, and poor transferability between simulated and experimental signals.

(3) In cross-domain damage localization tasks, a single loss function struggles to simultaneously accommodate both localization accuracy and cross-domain generalization performance simultaneously. Overemphasizing regression loss or lacking explicit constraints on higher-order statistical differences across domains often exacerbates conflicts between different optimization objectives.

To address these challenges, this study develops a digital twin-driven Lamb wave damage localization framework that integrates a two-stage adversarial transfer mechanism with Pareto-based multi-objective optimization. Existing DT-driven approaches typically treat cross-domain adaptation as parameter calibration or single-stage fine-tuning. In contrast, the proposed framework structurally separates digital-domain physics-guided feature enhancement from cross-domain adversarial alignment, forming a progressive and stable cross-domain learning paradigm. Digital simulated sensing data are first enriched to generate physically consistent and distribution-robust representations, which are subsequently aligned with experimental signals through a shared-encoder adversarial transfer network. A Pareto-based multi-objective strategy is further incorporated to dynamically coordinate localization accuracy, cross-domain robustness, and higher-order feature consistency, thereby mitigating conflicts among competing objectives. Extensive validation under diverse damage scenarios and extreme conditions confirms the framework’s robust generalization capability and reliable localization performance with scarce sensing data. The main contributions are summarized as follows:

(1) A digital model of electromechanically coupled guided waves based on piezoelectric excitation and sensing has been developed. This model achieves closer consistency with the experimental system in terms of excitation mechanisms, physical response quantities, and wave propagation behavior. The introduction of a perfectly matched layer (PML) suppresses boundary reflections, enabling the digital-domain signal to closely approximate the physical response in terms of energy attenuation and propagation characteristics.

(2) A two-stage adversarial and transfer-based digital twin framework for structural damage monitoring is proposed. Physics-informed hierarchical feature enhancement and conditional adversarial strategies enrich digital domain sensing data, while a lightweight transfer network based on BiGRU (bidirectional gated recurrent unit) ensures alignment with physical domain sensing data. This integration enables accurate damage localization even in the absence of experimental labels.

(3) The regression loss, domain adversarial loss, and multi-kernel maximum mean discrepancy (MK-MMD) regularization term are unified within an optimization framework, with dynamic trade-offs achieved using Pareto multi-objective optimization. This strategy effectively enhances the model’s localization stability and generalization capability across diverse damage scenarios and extreme conditions.

The remaining sections of this paper are organized as follows: Section 2 introduces the proposed DT-driven damage localization method. Section 3 details the case study used to validate the approach. Section 4 presents a comprehensive discussion of the results. Finally, Section 5 concludes the article.

## 2. Methodology

**Figure 1:**
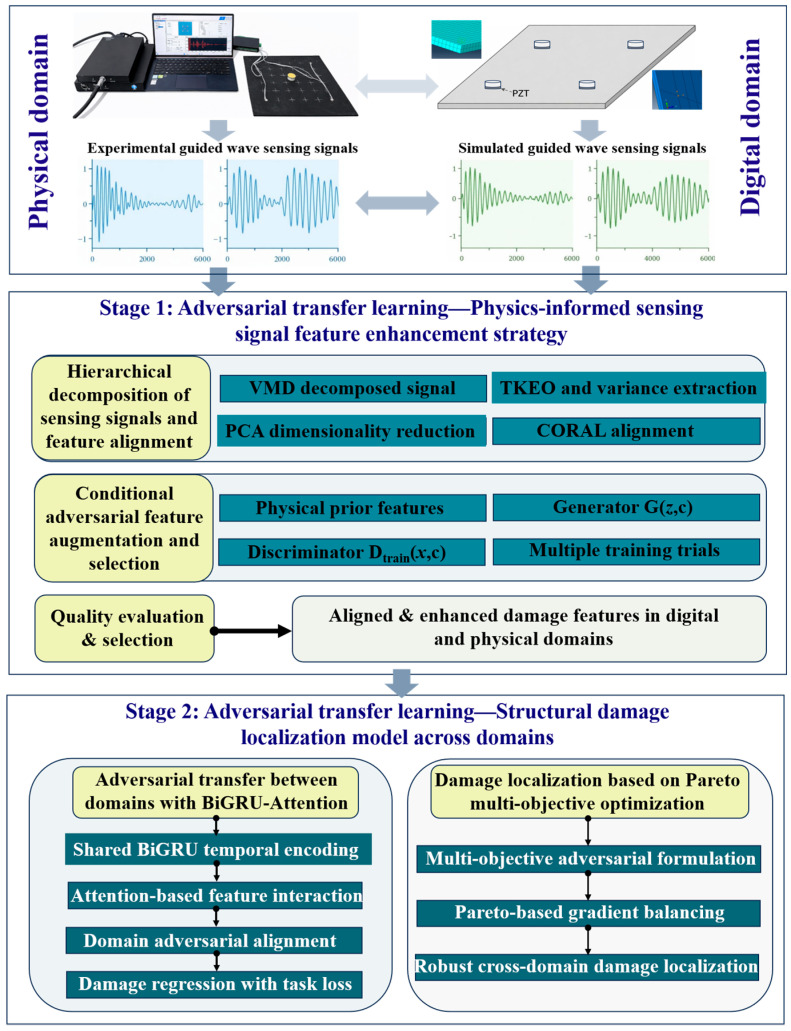
Schematic diagram of the holistic approach.

This study proposes a digital twin-driven dual-stage adversarial and transfer learning method with multi-objective optimization (DT-DSATMO) for Lamb wave-based structural damage localization under limited sensing conditions. The framework is illustrated in Figure 1. The digital domain refers to the simulation domain, where sensing data were generated using physics-based finite element modeling, whereas the physical domain denotes the experimental domain in which sensing signals were acquired from real structures using PZT sensors. Physics-based simulations generated digital-domain Lamb wave sensing data, which were first enhanced using physics-informed adversarial feature learning and then aligned with experimental sensing signals using a lightweight domain-adversarial network. A multi-objective optimization strategy ensured accurate and robust cross-domain localization.

### 2.1. Digital Modeling of PZT-Based Guided Wave Sensing Signals

Guided wave dispersion in laminated structures was first analyzed using a semi-analytical finite element approach, providing the basis for selecting excitation frequencies and propagation modes. Lamb wave propagation in carbon fiber-reinforced plastic (CFRP) plates was then simulated with an electromechanically coupled FEM. To suppress boundary reflections, the PML was applied, yielding a medium-fidelity digital model that generated rich PZT sensing signals.

#### 2.1.1. Dispersion Analysis of Guided Waves

Accurate structural health monitoring requires the reliable identification of Lamb wave modes, which is complicated by the anisotropic and laminated nature of CFRP plates. Guided wave dispersion plays a central role in this process, as it informs the selection of excitation frequency, mesh resolution, and time step to ensure physically consistent simulations.

The semi-analytical finite element (SAFE) method is applied to evaluate guided-wave dispersion in composite plates [26]. The dynamic behavior of Lamb waves is represented by the two-dimensional wave equation,

(1) $$\rho \frac{\partial^{2}u(x,y,z,t)}{\partial t^{2}}=\nabla \cdot \left[C:\nabla u\left(x,y,z,t\right)\right]$$

where u(x,y,z,t) denotes displacement, ρ is the material density, and C is the elastic constant matrix. The propagation direction (*x*-axis) is treated analytically, while the *y-z* plane is discretized using FEM, transforming the three-dimensional wave problem into a two-dimensional eigenvalue problem.

The phase and group velocities of the fundamental S_0_ and A_0_ modes were obtained as functions of frequency using the SAFE method. The group velocities describe the energy propagation speed and are used to determine the maximum allowable time step in the explicit dynamic simulations, ensuring numerical stability and accurate capture of guided wave propagation. Phase and group velocities are calculated according to Equations (2) and (3), respectively,

(2) $$v_{p}=\frac{\omega \cdot \lambda_{wave}}{2\pi }=F\cdot \lambda_{wave}$$

(3) $$v_{g}=\frac{d\omega }{dw}=v_{p}^{2}{\left[v_{p}-(FH)\frac{dv_{p}}{d(FH)}\right]}^{-1}$$

where F denotes the excitation center frequency, H is the plate thickness, and FH is the frequency–thickness product. The resulting dispersion curves are subsequently used in both simulated and experimental signal analyses for reliable damage localization.

#### 2.1.2. Finite Element Simulation of Guided Wave Propagation

After calculating the dispersion characteristics, this study employed FEM to simulate Lamb wave propagation in laminated plates, enabling the coupled electromechanical field to be solved under various boundary conditions. First, a finite element model of the CFRP plate was established, with PZT incorporated as both the actuators and sensors. The actuators apply voltage signals at specific frequencies to generate guided waves, while the sensors capture waveform variations during propagation, allowing for an analysis of features such as damage-induced reflections and attenuation.

The simulation is based on a set of commonly used partial differential equations describing the mechanical and electrical behavior of the material, as given in Equations (4) and (5),

(4) $$\sigma_{ij,j}+b_{i}=\rho a_{i},\;in\;\Omega^{S+P}$$

(5) $$D_{i,i}=0,\;in\;\Omega^{P}$$

where Equation (4) represents momentum conservation, accounting for variations in density, acceleration, body forces, and the stress tensor. Equation (5) ensures electric flux conservation in the absence of free charges, governing electromechanical behavior in PZT regions. In these equations, $\sigma_{ij}$ denotes stress, $b_{i}$ is body force, $d_{i}$ is displacement, $a_{i}$ is acceleration, and $D_{i}$ is electric displacement. The domains are specified as $\Omega^{P}$ for the PZT and $\Omega^{S+P}$ for the PZT, adhesive, and plate.

To capture guided-wave propagation accurately, the simulation incorporates boundary conditions, including displacement, traction, and electric potential constraints, as detailed in Equations (6)–(8) [27],

(6) $$a_{i}=\bar{a_{i}}\;and/or\;\sigma_{ij}n_{i}=\bar{c_{i}},\;on\;\Omega^{S+P}$$

(7) $$\emptyset =\bar{V}\;and/or\;D_{i}n_{i}=\bar{Q},\;on\;\Omega^{P}$$

(8) $$a\left(c=0\right)=a_{0}\;and\;\dot{a}\left(c=0\right)=v_{0},\;in\;\partial \;\Omega^{S+P}$$

where Equation (6) represents displacement or surface traction boundary conditions, Equation (7) defines electric potential or charge boundary conditions, and Equation (8) specifies initial displacement and velocity. The material constitutive relations describe the coupled mechanical and electrical behavior of guided waves in the CFRP plate, as expressed in Equations (9) and (10), where stress is coupled with the strain and electric displacement with the electric field,

(9) $$\sigma =C\varepsilon -e^{T}E$$

(10) $$D=e\varepsilon -\varepsilon_{\theta }E$$

where ε denotes the strain tensor, e is the piezoelectric coupling matrix, E is the electric field, and $\varepsilon_{\theta }$ is the dielectric constant under constant mechanical strain, consistent with standard piezoelectric constitutive modeling.

However, finite computational domains in FEM lead to boundary reflections that distort results. To address this, the PML is introduced around the composite plate, serving as an absorbing boundary that increases damping and eliminates reflections, thereby preserving the reliability of the simulation [28].

The PML region is implemented using an exponentially increasing Rayleigh damping model, with its damping matrix defined as follows,

(11) R=αA+βB

where A and B denote the mass and stiffness matrices, respectively, while α and β are the damping coefficients. α increases exponentially along the spatial coordinate *x* as follows,

(12) $$\alpha (x)=\alpha_{0}\cdot {\left(\frac{x}{l}\right)}^{o}$$

where *x* represents the local coordinate measured from the boundary of the main domain into the PML, l is the PML thickness, $\alpha_{0}$ denotes the maximum damping coefficient, and o is the decay rate exponent, which is set to 3.

### 2.2. Physics-Informed Sensing Signal Feature Enhancement Strategy

**Figure 2:**
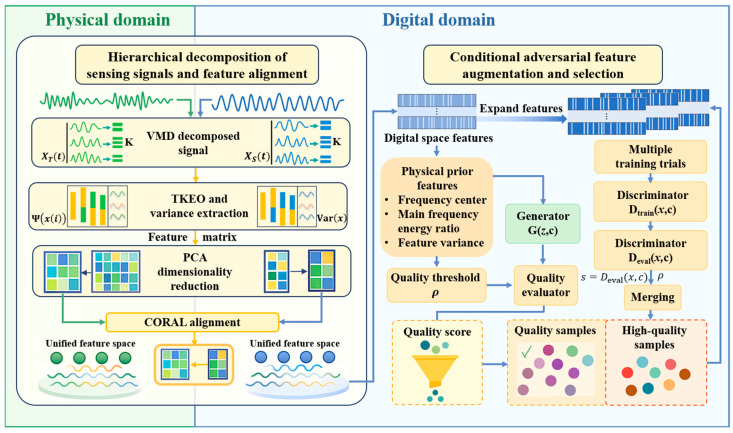
Stage 1: physics-informed feature enhancement in the adversarial transfer framework.

This section describes the first stage of the two-stage adversarial transfer framework, introducing a physics-guided sensing signal feature enhancement strategy, as illustrated in Figure 2. By incorporating Lamb wave propagation and energy distribution priors into feature decomposition, statistical characterization, distribution alignment, and conditional adversarial generation, the framework produces digital domain PZT sensing features that maintain strong physical consistency and cross-domain transferability, forming a robust basis for damage localization.

#### 2.2.1. Hierarchical Decomposition of Sensing Signals and Feature Alignment

Lamb wave signals contain multiple frequency components, noise, as well as structural reflections, often obscure subtle damage features. A hierarchical feature processing framework is proposed, integrating signal decomposition, feature extraction, dimensionality reduction, and cross-domain alignment to achieve unified feature representations between digital and experimental PZT signals.

Variational mode decomposition (VMD) is employed to decompose the original signals $X_{S}(t)$ and $X_{T}(t)$ into a set of band-limited intrinsic mode functions (IMFs). Each IMF $g_{k}$ is associated with a center frequency $h_{k}$, determined adaptively to ensure finite bandwidth [29]. The optimization model of VMD is formulated in Equation (13),

(13) $$\underset{\left\{g_{k}\right\},\{h_{k}\}}{\min}\sum_{k=1}^{K}{\left\|\gamma_{t}\left[\left(\delta \left(t\right)+\frac{j}{\pi t}\right)*g_{k}(t)\right]e^{-jh_{k}t}\right\|}_{2}^{2}$$

(14) $$s.t.\;\sum_{k=1}^{K}g_{k}(t)=f(t)$$

where ∗ denotes convolution, $\delta \left(t\right)$ is the impulse function, $\gamma_{t}$ the time derivative, and ${\left\|\cdot \right\|}_{2}^{2}$ the bandwidth measure. This problem is solved iteratively by updating IMFs and their center frequencies under the reconstruction constraint. IMFs with significant energy and damage relevance are retained, while noise-dominated modes are discarded, yielding analyzable components that preserve damage-related information and suppress high-frequency noise.

Subsequently, two damage-sensitive signal features were extracted from each IMF: the Teager–Kaiser energy operator (TKEO) and the variance. TKEO highlights instantaneous energy variations and amplifies subtle nonlinear transients, making it effective for detecting damage-induced disturbances:

(15) $$\Psi \left(x\left(i\right)\right)={x(i)}^{2}-x(i+1)\cdot x(i-1)$$

Variance quantifies signal fluctuations and is highly sensitive to low-energy variations, as calculated in Equation (16),

(16) $$Var(x)=\frac{1}{N-1}\sum_{i=1}^{N}{\left(x\left(i\right)-\bar{x}\right)}^{2}$$

where $\bar{x}$ denotes the mean of the signal. After computing these two types of features for each IMF, they were combined to form a joint feature vector, resulting in the initial feature matrices $\Gamma_{S}$ and $\Gamma_{T}$ for the digital and physical domains, respectively.

Although the extracted feature vectors contain rich damage-related information, they remain high-dimensional, which hinders cross-domain feature comparison. To address this, principal component analysis (PCA) was employed to reduce the dimensionality of data from both domains [30]. This process decreased redundancy and noise while ensuring that features from the two domains were compared within a unified subspace.

The original feature dimension is denoted as $\Pi_{max}$ and the reduced subspace dimension is ι. The PCA transformation can be expressed as Equation (17),

(17) $$Y_{S}=W_{S}^{T}\Gamma_{S},\;Y_{T}=W_{T}^{T}\Gamma_{T},$$

where $W_{S},W_{T}\in R^{\Pi_{max}\times \iota }$ are the projection matrices for each domain, obtained through the eigen-decomposition of the corresponding feature covariance matrices.

To mitigate the residual inter-domain distribution differences after PCA, this study employed the correlation alignment (CORAL) method, which aligns the covariance matrices of the source and target domains to achieve second-order statistical matching [31]. The covariance matrices of the two domains are given by Equation (18):

(18) $$O_{S}=cov\left(Y_{S}\right)+I,\;O_{T}=cov\left(Y_{T}\right)+I$$

CORAL obtains the transformation matrix A by solving the following optimization problem:

(19) $$\underset{A}{\min}{\left\|A^{T}O_{S}A-O_{T}\right\|}_{\Lambda }^{2}$$

where ${\left\|\cdot \right\|}_{\Lambda }$ denotes the Frobenius norm. In the closed-form solution, the transformation matrix is expressed as Equation (20):

(20) $$A=O_{S}^{-\frac{1}{2}}\cdot {\left(O_{S}^{\frac{1}{2}}O_{T}O_{S}^{\frac{1}{2}}\right)}^{\frac{1}{2}}\cdot O_{S}^{-\frac{1}{2}}$$

Finally, the digital domain features were mapped using this transformation to align their covariance with that of the physical domain, yielding the aligned features.

(21) $${\tilde{Y}}_{S}=Y_{S}A$$

Through the progressive processing of the above four steps, the Lamb wave signals from the physical and digital domains are ultimately mapped into a feature space that is noise-suppressed, physically relevant, and distributionally aligned. This approach preserves the damage sensitivity of both transient and statistical features while effectively mitigating inter-domain distribution discrepancies.

#### 2.2.2. Conditional Adversarial Feature Augmentation and Selection

Although feature distributions between the two domains are aligned, improving the diversity and quality of digital-domain samples remains essential for adapting to experimentally acquired PZT sensing data. A conditional adversarial feature augmentation and selection strategy was introduced in which physics-informed constraints guide adversarial generation while a discriminator-based mechanism filters out low-quality samples.

A conditional vector was constructed containing the central frequency, the dominant frequency energy ratio, and the feature variance. It ensured that the generated virtual Lamb wave features remained statistically consistent with the experimental data and physically representative of damage features. The central frequency $f^{\left(c\right)}$ is defined as in Equation (22),

(22) $$f^{\left(c\right)}=\frac{\sum_{m=1}^{M}f_{m}^{\left(sig\right)}\cdot {\left|X(f_{m}^{\left(sig\right)})\right|}^{2}}{\sum_{m=1}^{M}{\left|X(f_{m}^{\left(sig\right)})\right|}^{2}}$$

where $f_{m}^{\left(sig\right)}$ and $X(f_{m}^{\left(sig\right)})$ denote the discrete spectral components and their amplitudes, respectively. M represents the total number of discrete spectrum points.

The dominant frequency energy ratio reflects the proportion of signal energy concentrated around the central frequency band and is defined in Equation (23),

(23) $$\varphi_{r}=\frac{\sum_{f_{m}^{\left(sig\right)}\in \left[f^{\left(c\right)}-{∆}_{f}\right]}{\left|X(f_{m}^{\left(sig\right)})\right|}^{2}}{\sum_{m=1}^{M}{\left|X(f_{m}^{\left(sig\right)})\right|}^{2}}$$

where $\varphi_{r}$ denotes the principal frequency energy ratio and ${∆}_{f}$ represents the analysis bandwidth. The feature variance further characterizes the overall amplitude fluctuations of the signal. These physics-informed descriptors are incorporated into the conditional vector, which imposes strong constraints on the generator and ensures that the synthesized samples remain physically consistent with real damage responses.

In the adversarial framework, the generator $G\left(z,c\right)$ produces pseudo-samples conditioned on *c*, while the discriminator D(x,c) assesses authenticity using a Wasserstein score with gradient penalty. The generator was trained to maximize this score, as defined in Equation (24) [32]:

(24) $${Loss}_{G}^{cond}=-E_{\tilde{x}\sim P_{g}}[D(\tilde{x},c)]$$

To enhance the quality of the generated data, a sample filtering mechanism was introduced to retain only high-quality pseudo-samples based on the discriminator’s evaluation. The process operates in four steps:

(1) The generator outputs a set of pseudo-samples $\tilde{x}$, and the discriminator assigns each sample a score $s=D(\tilde{x},c)$.

(2) A quality threshold ρ is specified, and samples satisfying $D(\tilde{x},c)\geq \rho$ are preserved. The resulting set of accepted samples ${\tilde{x}}_{keep}$ is defined in Equation (25):

(25) $${\tilde{x}}_{keep}=\left\{\begin{array}{cc} {\tilde{x}}_{keep}, & if\;D(\tilde{x},c)\geq \rho \\ \mathrm{discard}, & \mathrm{otherwise} \end{array}\right.$$

Typically, ρ is chosen between 0.3 and 0.5 or adjusted to retain the top-K% of the highest-scoring samples. This filtering mechanism effectively removes low-quality outputs, thereby improving the validity and stability of the augmented dataset.

(3) To further enhance the diversity and robustness of the generated samples, a multi-model training and result fusion strategy was employed. The conditional WGAN-GP was trained repeatedly ($N_{trial}=3\sim 5$), generating *n* pseudo-samples in each trial.

(4) The high-quality samples obtained from each trial are filtered and then merged to form the final augmented dataset $X_{aug}$:

(26) $$X_{aug}=\overset{N_{trial}}{\underset{i=1}{⋃}}\left\{{\tilde{x}}_{keep}^{\left(i\right)}\right\}$$

Overall, this framework enhances the diversity and physical fidelity of simulated Lamb wave sensing data under limited experimental conditions, providing reliable virtual sensing signals for robust damage localization and identification.

### 2.3. Structural Damage Localization Model Across Domains

This section presents the second stage of the two-stage adversarial transfer framework. A lightweight domain-adversarial network aligns digital domain simulation features with unlabeled physical domain experimental features in a shared encoding space, using digital features as priors for regression and feature calibration. Pareto-based multi-objective optimization balances localization accuracy, domain invariance, and distribution consistency [33].

#### 2.3.1. Adversarial Transfer Between Domains with BiGRU-Attention

**Figure 3:**
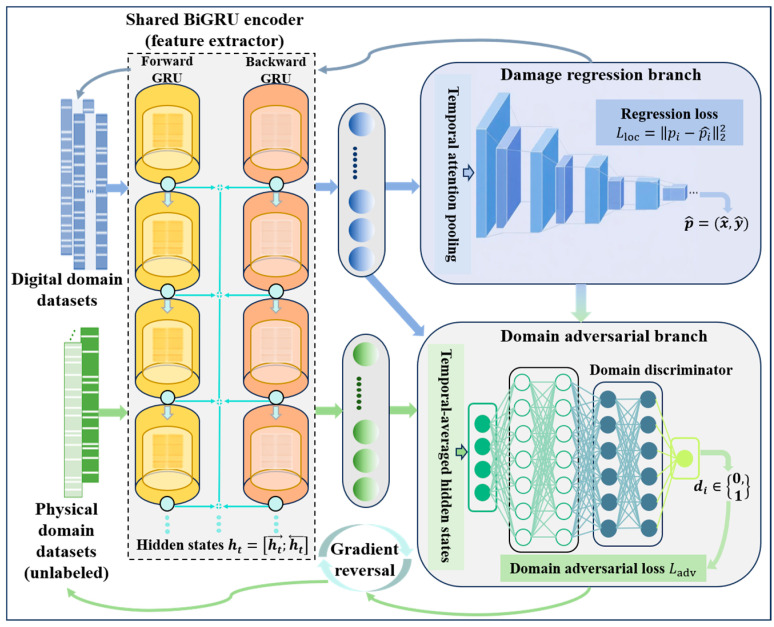
Stage 2: Lightweight BiGRU-Attention adversarial transfer network in the adversarial transfer framework.

The input to the BiGRU-Attention integrated adversarial transfer network consists of two types of aligned samples: digital domain samples and unlabeled physical domain samples. To address the issues of redundant computation and feature mismatch inherent in the traditional discrete feature extractor–regression head–domain discriminator structure, this study proposes a lightweight integrated design, as illustrated in Figure 3.

Specifically, the front layers of the BiGRU encoder extract temporal features from physics-informed representations of the digital domain and physical domain sensing signals, producing shared latent feature embeddings. These features are used for damage regression prediction on the one hand and are fed into a domain discriminator via a gradient reversal layer (GRL) on the other hand to enforce adversarial domain alignment. In this way, prediction accuracy and domain invariance are jointly achieved within a single encoding process. The detailed rules of the lightweight integrated design are provided in Algorithm 1.
**Algorithm 1: Hybrid BiGRU-Attention regression and domain-adversarial learning.****Input:**  Aligned digital domain $\mathrm{feature}\;\mathrm{set}\;D_{S}=\left\{\left(X_{S}^{i},p_{S}^{i}\right)\right\}$  Aligned physical domain $\mathrm{feature}\;\mathrm{set}\;D_{T}=\left\{\left(X_{T}^{j}\right)\right\}$ **Output:**  $\mathrm{Predicted}\;\mathrm{damage}\;\mathrm{coordinates}\;\hat{p}={\left[\hat{x},\hat{y}\right]}^{T}$**1.** Initialize BiGRU-Attention encoder F, regression head R, domain discriminator D, and GRL coefficient λ=0.**2.** **For each** training epoch:**3.** Encode the physics-informed sensing feature sequences using BiGRU in forward and backward directions, and concatenate hidden states to obtain temporal feature embeddings.**4.** Compute attention weights to highlight key guided-wave segments and aggregate them into a global sensing feature vector g via weighted sum.**5.** Predict damage coordinates using the regression head: p = R(g).**6.** Perform temporal averaging on BiGRU outputs to get domain feature $\bar{h}$, pass through GRL and the domain discriminator D to estimate domain labels.**7.** Compute regression loss $L_{reg}$ and domain loss $L_{dom}$ (binary cross-entropy).**8.** Update D by minimizing $L_{dom}$; update F and R by minimizing $L_{reg}+\lambda L_{dom}$.**9.** Reverse gradients through GRL and gradually increase λ to strengthen cross-domain sensing alignment.**10.** **End for.****11.** **Return** final prediction: $\hat{p}=f_{BiGRU-Attn}(X)$.

The interaction between the digital and physical domains is embedded in the training process through shared feature embeddings and adversarial learning. Labeled simulated sensing features provide regression supervision, guiding the encoder toward damage-discriminative representations, while physical domain features convey cross-domain distribution information via the domain discriminator, enabling iterative correction of domain shifts. This closed-loop design allows for the encoder to jointly capture and align statistical differences between virtual and real sensing signals.

In summary, the proposed network achieves tight integration of regression supervision and domain-adversarial learning. It reduces feature fragmentation and redundant computation, efficiently captures fine-grained patterns of damage-induced wave propagation, and significantly improves damage localization accuracy and cross-domain robustness within a lightweight framework.

#### 2.3.2. Pareto-Optimized Damage Localization Using Sensing Features

**Figure 4:**
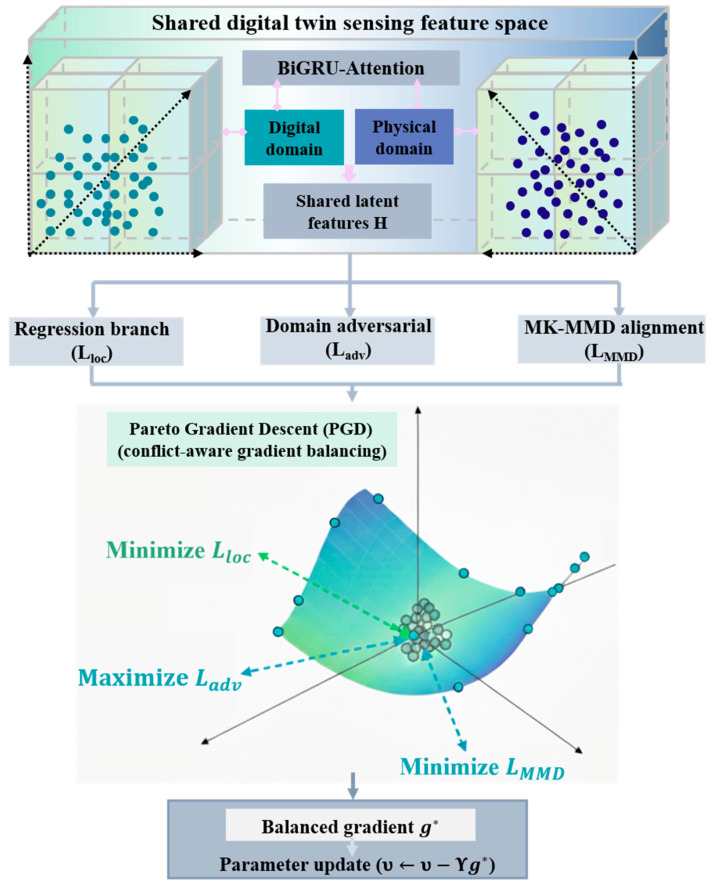
Schematic of the multi-objective optimization mechanism.

The network described in the previous section outputs the predicted damage location coordinates in the form $\hat{p}={\left[\hat{x},\hat{y}\right]}^{T}$. However, relying on a single loss function for training often fails to balance localization accuracy and cross-domain robustness. To address this, the present study introduces a multi-objective optimization strategy that jointly incorporates the regression loss $L_{loc}$, the domain adversarial loss $L_{adv}$, and the MK-MMD regularization term $L_{MMD}$ into the training objective, forming a collaborative optimization mechanism, as shown in Figure 4.

First, the localization regression loss is defined using the mean squared error (MSE), as shown in Equation (27),

(27) $$L_{loc}=\frac{1}{N_{s}}\sum_{i=1}^{N_{s}}{\left\|p_{i}-\hat{p_{i}}\right\|}_{2}^{2}$$

where $p_{i}$ denotes the true coordinates of the digital domain sample, $\hat{p_{i}}$ represents the predicted coordinates, and $N_{s}$ is the number of digital domain samples.

Subsequently, the domain adversarial loss is defined based on the cross-entropy function, as shown in Equation (28),

(28) $$L_{adv}=-\frac{1}{N}\sum_{i=1}^{N}\left[d_{i}\log\left(\hat{d_{i}}\right)+(1-d_{i})\log\left(1-\hat{d_{i}}\right)\right]$$

where $d_{i}\in \left\{0,1\right\}$ denote the domain label, $\hat{d_{i}}$ is the predicted probability from the domain classifier, and *N* is the total number of training samples.

Finally, MK-MMD is introduced to constrain higher-order distribution consistency between the two domains,

(29) $$L_{MMD}=\sum_{m=1}^{M}\sigma_{m}{\left\|\frac{1}{N_{s}}\sum_{i=1}^{N_{s}}\psi_{m}(x_{i}^{s})-\frac{1}{N_{t}}\sum_{j=1}^{N_{t}}\psi_{m}(x_{j}^{t})\right\|}_{H_{m}}^{2}$$

where $\psi_{m}$ denotes the feature mapping induced by the kernel function $k_{m}(\cdot ,\cdot )$. $\sigma_{m}$ is the corresponding kernel weight. $N_{s}$ and $N_{t}$ represent the numbers of digital domain and physical domain samples, respectively. This term enhances the fine-grained alignment of feature distributions between the two domains.

Integrating the above three objectives, the combined loss can be expressed as Equation (30),

(30) $$\underset{\varpi_{\mathrm{enc}},\varpi_{\mathrm{reg}}}{\min}\underset{\varpi_{\mathrm{dom}}}{\max}\left(L_{loc}+pL_{adv}+qL_{MMD}\right)$$

where $\varpi_{enc},\varpi_{reg}$, and $\varpi_{dom}$ denote the parameters of the feature extractor, regressor, and domain classifier, respectively, and p and q are the corresponding loss weight coefficients.

However, simple weighted-sum optimization may cause unstable convergence due to gradient conflicts. To address this issue, Pareto gradient descent (PGD) was employed. Gradients for each loss are computed as in Equation (31),

(31) $$g_{loc}=\nabla_{\upsilon }L_{loc},\;g_{adv}=\nabla_{\upsilon }L_{adv},g_{MMD}=\nabla_{\upsilon }L_{MMD}$$

where υ denotes the parameter to be optimized. PGD seeks a Pareto-optimal gradient $g^{*}$ that balances conflicts among tasks.

(32) $$g^{*}=\sum_{i}u_{i}g_{i},\;s.t.\;u_{i}\geq 0,\;\sum_{i}u_{i}=1$$

where $g_{i}\in \left\{g_{loc},g_{adv},g_{MMD}\right\}$ and the coefficient $u_{i}$ are determined by solving a quadratic programming problem to ensure Pareto-optimality. Model parameters are updated using this balanced gradient,

(33) $$\upsilon \leftarrow \upsilon -\Upsilon g^{*}$$

where Υ denotes the learning rate.

PGD dynamically balances localization, domain-adversarial, and distribution objectives, reducing training oscillations and improving the robustness and accuracy of cross-domain damage localization.

## 3. Case Study

A typical aircraft wing-box panel component was used to validate the proposed method within a digital twin-based structural health monitoring framework. The component consisted of a square CFRP specimen with a side length of 280 mm and a uniform thickness of 2 mm, fabricated from T700 12 K unidirectional carbon fiber prepregs. The mechanical properties of the material were provided by the manufacturer and are listed in Table 1.

### 3.1. Experiment Sensor Signal Acquisition in Physical Domain

**Figure 5:**
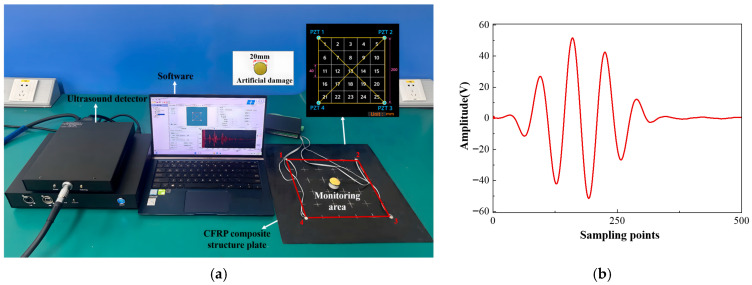
(**a**) Experimental platform and (**b**) five-cycle tone-burst excitation.

An experimental platform for CFRP damage monitoring was established based on ultrasonic guided wave sensing. The setup includes the CFRP plate, a PZT sensor array, an ultrasound detector, and a control computer. A 200 mm × 200 mm central area of the plate was defined as the monitoring region. Four PZT sensors with a diameter of 8 mm and a thickness of 0.45 mm were mounted on the plate’s surface, forming six effective excitation–reception paths for multi-path guided wave sensing, as illustrated in Figure 5a. For systematic analysis, the monitoring area was uniformly divided into 25 regions, each representing a potential damage site.

**Figure 6:**
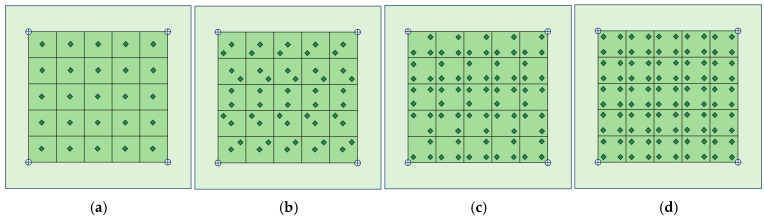
Experimental training datasets collected for multiple damage locations ((**a**–**d**) represent four datasets).

The experiment employs a five-cycle sinusoidal wave with a Hanning window and a central frequency of 150 kHz as the excitation signal, as illustrated in Figure 5b. This signal was applied to the excitation PZT sensor using the ultrasonic guided wave monitoring system while the remaining PZT sensors simultaneously acquired the propagated Lamb wave responses. The excitation voltage was set to 150 V, and the received signals were amplified by 40 dB and sampled at 24 MS/s to ensure sufficient time-domain resolution and preservation of signal features. Model defects were introduced on the CFRP composite plates using a tacky, wave-absorbing material to create artificial damage. This approach generated localized changes in structural stiffness and wave energy absorption, which induced variations in guided wave propagation, including phase shifts and amplitude attenuation, similar to those caused by real delamination [34,35]. The circular artificial damage with a diameter of 20 mm and a thickness of approximately 4 mm was applied at various locations on the plate’s surface. By varying the damage locations within the monitoring area, Lamb wave responses under different damage scenarios were obtained, resulting in four experimental datasets for training, as shown in Figure 6.

### 3.2. Simulated Sensor Signal Generation in Digital Domain

#### 3.2.1. Signal Simulation in Healthy State

This study constructs a medium-fidelity finite element model of PZT-based Lamb wave propagation in CFRP composite plates. All simulations were performed in ABAQUS 6.12. The numerical stability in the guided wave simulations is ensured based on the dispersion analysis presented in Section 2.1.1. Specifically, the SAFE-based dispersion study provided the group velocities of the fundamental S_0_ and A_0_ Lamb wave modes, which were used to determine the spatial and temporal resolution requirements. The mesh size was chosen to resolve at least 8 elements per wavelength for each mode, ensuring adequate spatial resolution in accordance with the dispersion characteristics. The time increment was selected to satisfy both the waveform resolution criterion (≥20 steps per wave period) and the Courant–Friedrichs–Lewy condition, with the maximum allowable step size determined by the fastest propagating mode [36,37]. An explicit dynamic solver was employed with a fixed time increment of 0.1 μs, thereby maintaining numerical stability while accurately capturing the guided wave propagation in the CFRP plate.

**Figure 7:**
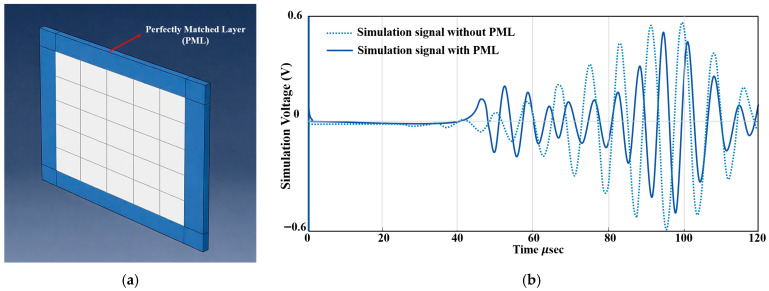
Configuration and validation of the PML. (**a**) Schematic configuration of the PML applied around the plate boundary, and (**b**) comparison of simulated sensor signals with and without PML implementation.

In the finite element model, only boundary reflection attenuation is considered, as it has the most significant impact on the extraction of damage features and the reliability of damage localization results. A perfectly matched layer (PML) is applied around the plate to reduce boundary reflections, as illustrated in Figure 7a. The PML gradually increases damping along the boundary, effectively absorbing outgoing wave energy and suppressing nonphysical reflections. The key parameters of the PML, including its thickness and maximum damping coefficient, are determined based on the characteristic wavelengths of the guided wave modes at the excitation frequency. The excitation frequency is 150 kHz, at which the group velocities of the fundamental S_0_ and A_0_ Lamb wave modes in the CFRP plate are 6758.7 m/s and 1556 m/s, respectively. These values correspond to wavelengths of approximately 45.1 mm for the S_0_ mode and 10.4 mm for the A_0_ mode. Since the S_0_ mode exhibits a longer wavelength and is more difficult to attenuate, the PML thickness is selected based on the S_0_ wavelength. A PML thickness of 80 mm, corresponding to approximately 1.8 times the dominant wavelength, is adopted to ensure the sufficient attenuation of outgoing guided waves. In addition to the PML thickness, the maximum damping coefficient α_0_ is determined through preliminary numerical tuning by monitoring the decay of wave amplitudes within the PML. α_0_ is chosen such that reflected wave energy at the interface between the PML and the physical domain is negligible, while avoiding over-damping that could affect wave propagation within the main computational region.

Figure 7b further illustrates the effectiveness of the PML by superimposing simulated sensor signals obtained with and without PML implementation. At early time instants (0–40 μs), the two simulated signals exhibit nearly identical waveforms, indicating that the PML does not distort the incident Lamb wave propagation within the main computational domain. As time progresses, noticeable discrepancies emerge in the absence of the PML. In particular, pronounced late-arriving wave packets with increased amplitude are observed after approximately 70 μs, which can be attributed to spurious boundary reflections caused by the finite computational domain. By contrast, these nonphysical reflections are effectively suppressed when the PML is applied, resulting in a cleaner signal that contains only physically meaningful wave components. This comparison confirms that the adopted PML configuration provides efficient absorption of outgoing guided waves and ensures reliable numerical signal generation.

**Figure 8:**
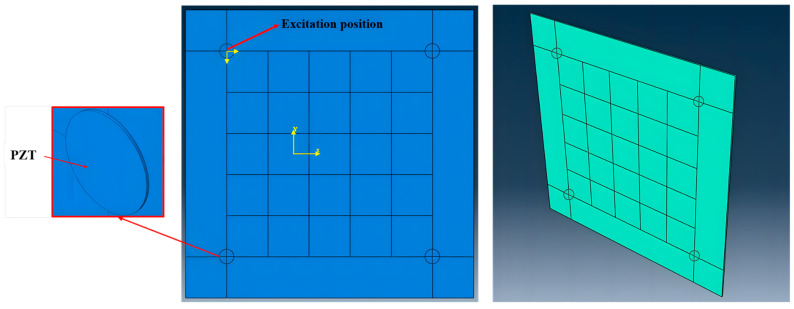
Finite element model of PZT actuation–sensing electromechanical coupling.

To ensure consistency between the simulation and experiments regarding the excitation and sensing mechanisms, this study employed a PZT-based electromechanical coupling finite element modeling strategy. Departing from conventional simplified models reliant on point excitation and node displacement, the proposed method incorporates sensing mechanisms. As illustrated in Figure 8, the PZT sensors concurrently perform wave-guided excitation and signal sensing functions. It converts applied voltage signals into mechanical strain fields and transforms structural vibration responses back into voltage signals. This modeling approach not only authentically reflects the electromechanical coupling effects within piezoelectric materials, but also enables direct comparison between simulated voltage outputs and experimentally acquired signals, thereby eliminating dimensional inconsistencies at the source. The dimensions of the PZT sensors are consistent with those used in the experiments, and the sensors are bonded to the surface of the CFRP plate using a 0.05 mm thick adhesive layer. The CFRP plate geometry is likewise consistent with the experimental specimen. Tie constraints ensured reliable connections between the PZT, adhesive layer, and plate structure. The material parameters are detailed in Table 2.

The CFRP plate model utilizes hyperbolic four-node shell elements for discretization, and the PZT sensor is modeled using three-dimensional C3D8E solid elements that incorporate electromechanical coupling, with a mesh size of 0.5 mm. The CFRP plate is discretized using doubly curved four-node shell elements, while the PZT patches are modeled with three-dimensional C3D8E solid elements incorporating electromechanical coupling, with a mesh size of 0.5 mm. The adhesive layer is represented using C3D8R elements with a mesh size of 0.2 mm. After defining the material properties, boundary conditions, and electrical excitation, an implicit dynamic analysis was performed to simulate the excitation, propagation, and sensing of Lamb waves. This modeling framework produces PZT sensing responses that are directly comparable to experimentally measured signals.

**Figure 9:**
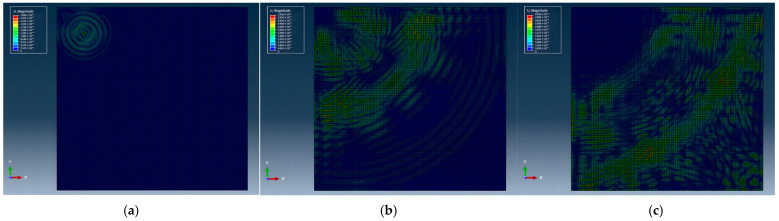
Propagation cloud maps of Lamb waves in the structure at different time instants. (**a**) t = 1.98 × 10^−5^ s, (**b**) 5.61 × 10^−5^ s, and (**c**) 9.24 × 10^−5^ s.

To visually demonstrate the propagation and evolution of Lamb waves within the structure, Figure 9 presents propagation cloud maps at three distinct time points. It can be observed that, following excitation of the piezoelectric element, Lamb waves gradually propagate from the excitation region into the plate. As the propagation distance increases, the interaction between incident and reflected waves produces progressively complex interference patterns, giving rise to new wave packets. At the same time, the overall response amplitude decreases due to material damping, geometric spreading, and boundary interactions.

**Figure 10:**
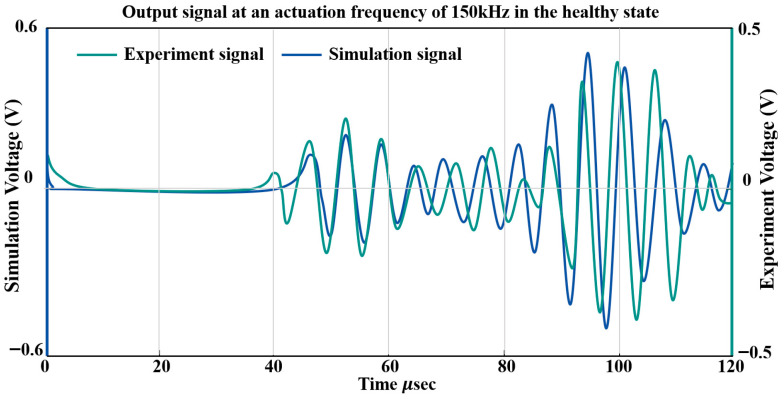
Comparison of simulated and experimental guided wave signals in the healthy state.

Figure 10 presents a comparison between the simulated and experimentally acquired PZT signals along the PZT2–PZT4 sensing path. The experimental signals undergo front-end amplification, so their absolute amplitudes are not directly comparable. Therefore, the analysis focuses on the group velocities of the guided-wave modes. The first arriving wave packet, corresponding to the S_0_ mode, shows strong agreement between the simulation and experiments. In contrast, later wave packets associated with the A_0_ mode display minor differences in arrival time and waveform characteristics, which are likely attributable to boundary reflections and mode-coupling effects.

#### 3.2.2. Signal Simulation in Damaged State

**Figure 11:**
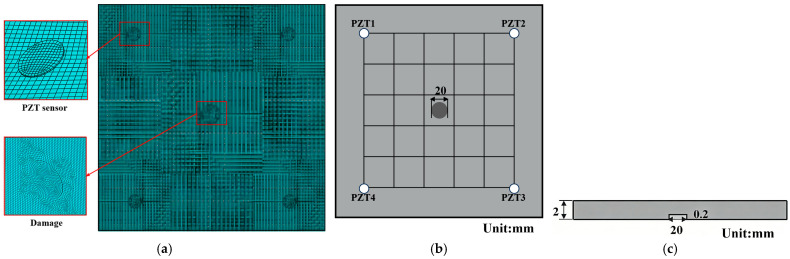
Schematic diagram of the damaged structure simulation. (**a**) FE meshing in the damaged state. (**b**) Top view of damaged structure. (**c**) Cross-sectional view of damaged structure.

**Figure 12:**
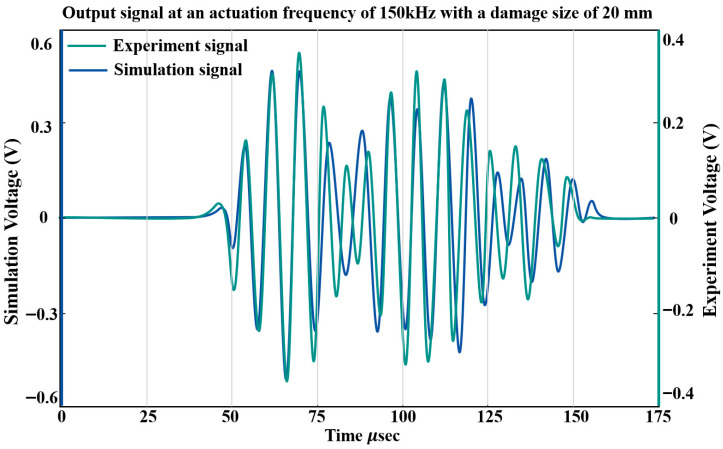
Comparison of simulated and experimental guided wave signals for a damage size of 20 mm.

For the damaged structure, delamination was explicitly modeled using a volume split approach in which a planar geometric discontinuity was introduced between two adjacent element layers in the damaged region. Figure 11a shows the finite element mesh of the structure containing the simulated damage. Specifically, a representative circular delamination defect with a diameter of 20 mm and a depth of 0.2 mm was introduced at the center of the composite structure, located between the top ply and the adjacent layer. To clearly illustrate the geometric configuration of the damage, a top view and a cross-sectional view of the delaminated region are provided in Figure 11b and Figure 11c, respectively. The volume split operation physically separates the corresponding finite elements across the predefined interface, thereby eliminating interlaminar stiffness and allowing for relative displacement between the layers, which effectively captures the mechanical characteristics of the delamination defects. All other material properties, stacking sequences, and boundary conditions remain identical to those of the undamaged model to ensure that the observed wavefield variations are solely induced by the introduced damage. Figure 12 compares the simulated and experimentally acquired PZT sensing signals for a damage size of 20 mm. The first wave packet shows good agreement between the simulated and experimental signals, whereas the second wave packet exhibits noticeable discrepancies due to structural boundary reflections and multimodal coupling. To address these residual differences between the simulated and experimental sensing data, a two-stage adversarial and transfer learning strategy was applied in the subsequent analysis to further reduce cross-domain distribution shifts.

**Figure 13:**
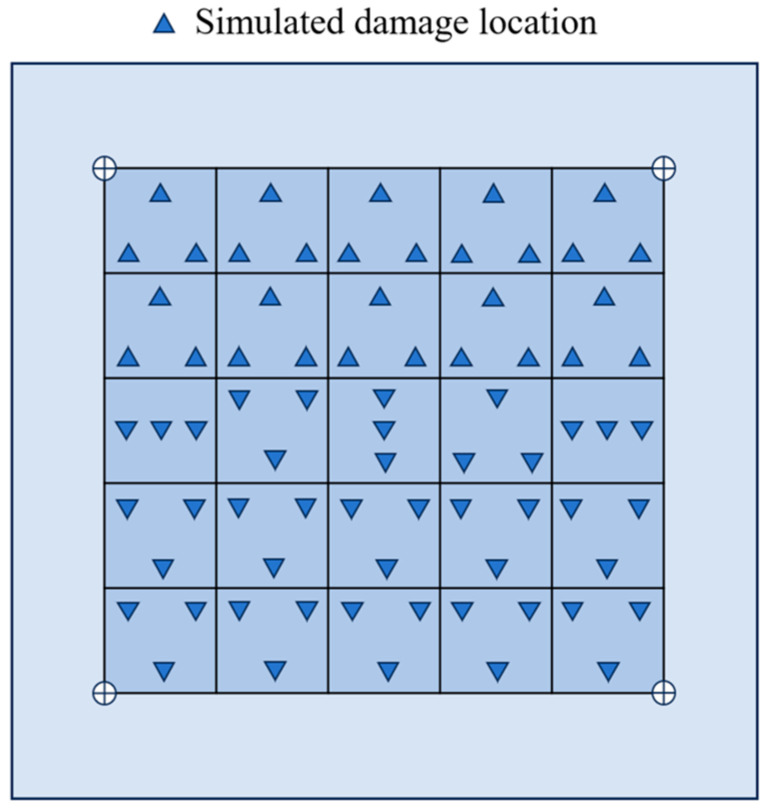
Sample distribution of the simulated training dataset.

A simulated training dataset of PZT-based Lamb wave sensing signals was constructed using the previously established damage model, with the sample distribution being illustrated in Figure 13. Triangles indicate simulated damage locations. By dividing the central monitoring area into 25 evenly spaced sub-regions and simulating three distinct damage locations within each sub-region, a total of 75 samples were generated. These samples served as the initial dataset for conditional adversarial feature augmentation and selection. This procedure ultimately produced a simulated training set comprising 400 damage samples. All numerical simulations were performed using a quad-core Intel i7-6500U CPU running at 2.5 GHz. Under this computational setup, the average simulation time for each damage scenario was approximately 2975 ± 621.4 s.

### 3.3. Baseline Methods for Comparative Study

To comprehensively evaluate the proposed DT-DSATMO framework, three representative baseline methods were implemented for comparison, including two classical physics-driven approaches, namely the delay-and-sum method (DAS) and the elliptical probability imaging method (EPI), as well as a conventional convolutional neural network (CNN)-based end-to-end localization model.

As for the DAS approach, the expected time-of-flight from the actuator to each candidate location and then to the receiver was calculated using the group velocity at 150 kHz, and the measured signals were time-shifted and coherently summed across all sensing paths. The grid point corresponding to the maximum reconstructed intensity was taken as the predicted damage location. In the EPI method, an elliptical locus was constructed for each actuator–sensor pair based on the time-of-flight difference between baseline and damage signals, and spatial probability distributions from all sensing paths were superimposed to generate the final damage probability map, where the point with the highest accumulated probability was identified as the localization result. In addition, a CNN-based end-to-end model was implemented to directly map preprocessed guided wave signals to damage coordinates with stacked one-dimensional convolutional layers followed by fully connected regression layers. Rectified linear unit (ReLU) activation functions were used, and the network was trained using the mean squared error (MSE) loss with the Adam optimizer.

For all methods, identical sensor configurations, excitation parameters, damage scenarios, signal preprocessing procedures, dataset partitions, and evaluation metrics were adopted to ensure a fair comparison.

## 4. Discussion

This section presents a case study analyzing the feature distributions of PZT-based Lamb wave sensing signals in both the digital and physical domains. It also provides a comparative evaluation of the damage monitoring performance across different scenarios and model configurations while validating the generalization capability of the proposed method under extreme conditions.

### 4.1. Feature Distributions Across the Two Domains

**Figure 14:**
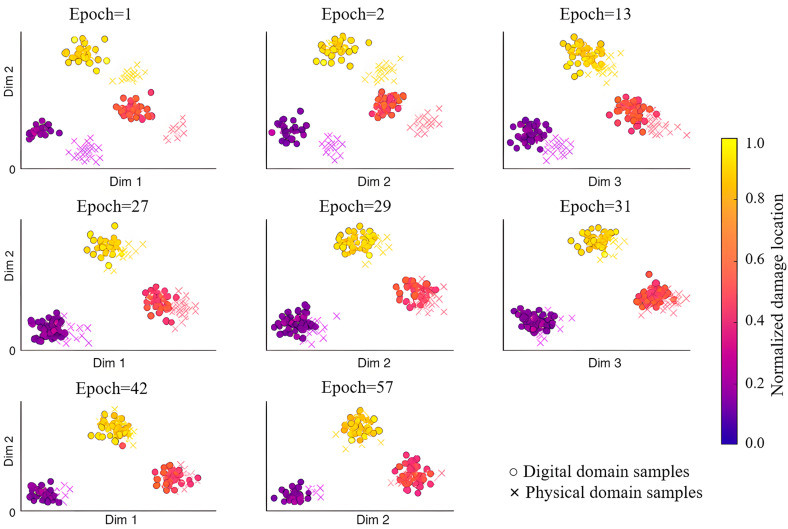
T-distributed stochastic neighbor embedding (t-SNE) visualization of cross-domain feature alignment at different training stages.

To illustrate the alignment of features across digital and physical domains and the mapping of damage locations at different training stages, t-distributed stochastic neighbor embedding (t-SNE) was employed to visualize the low-dimensional features extracted by the model at selected epochs, as shown in Figure 14. The horizontal and vertical axes represent the two principal components obtained from t-SNE. Symbols “○” denote the digital domain samples, and “×” denote the physical domain samples. Colors encode the normalized damage location coordinates, ranging from purple to yellow to indicate positions from left to right along the plate.

At the initial training stages (e.g., epoch 1 and 2), the features of samples from the two domains were clearly separated, indicating a pronounced distribution shift. The disordered and discontinuous color distribution indicates that the model did not yet establish stable feature mapping relationships, resulting in strong inconsistencies in damage location information across domains. This phase primarily reflects the supervisory role of the simulated sensor data, since unlabeled experimental samples were not yet effectively exploited for feature adaptation.

During subsequent training periods (e.g., 13, 27, 29, and 31), features from the two domains gradually converged. Samples from similar injury locations began to cluster and overlap across both domains, indicating that cross-domain alignment was progressively established through adversarial training and multi-objective optimization. This improvement benefits from the temporal dependency features extracted using the BiGRU-Attention module and the adversarial constraints imposed by the GRL. In later training epochs (e.g., epochs 42 and 57), samples of different colors exhibited near-complete overlap, with smooth and continuous color transitions across domains. This demonstrates that the model achieved cross-domain feature alignment while preserving damage location information.

**Figure 15:**
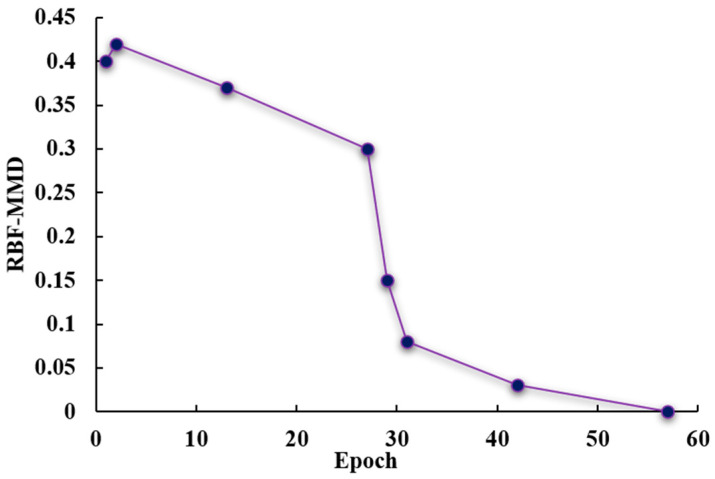
Evolution of RBF-MMD between digital and physical domains during training.

Overall, t-SNE reveals a clear evolution from inter-domain separation to feature fusion, indicating that the two-stage adversarial transfer framework combined with multi-objective optimization effectively promotes cross-domain consistency in feature distributions. Furthermore, this study employed the maximum mean discrepancy based on a radial basis function kernel (RBF-MMD) to quantify inter-domain differences, as shown in Figure 15. Its monotonic decrease during training demonstrates enhanced the invariance of the sensing feature domain and improved the regression accuracy on the experimental sensing data.

### 4.2. Damage Localization Under Various Scenarios

To systematically assess the generalization capability and damage localization accuracy of the proposed method under different cross-domain sensing conditions, six damage diagnosis tasks (S1–S6) were designed. These tasks took into account the abundance of samples in the digital domain and the limited availability of labeled samples in the physical domain. Five damage locations were randomly selected in each scenario, with the detailed configuration of each task being summarized in Table 3. The localization models were implemented in Python (version 3.6.13) using the Keras framework (version 2.3.1), and training was conducted on a system equipped with a 12-core Intel i5-10400 CPU and 8 GB of RAM.

**Figure 16:**
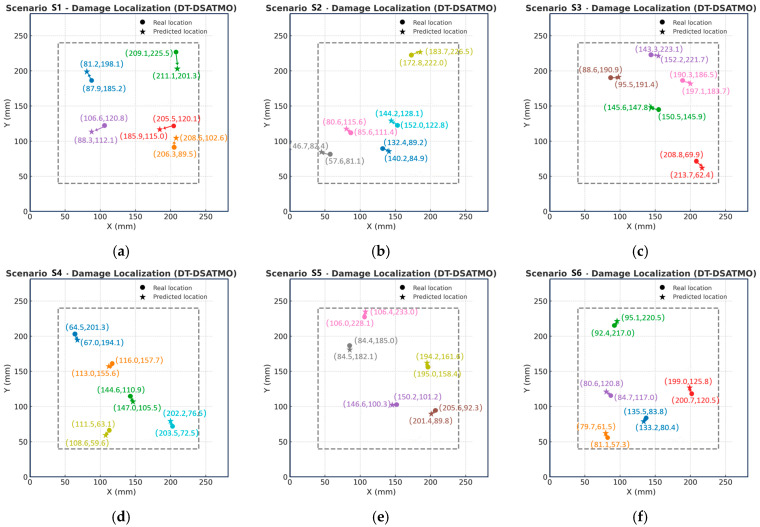
Damage localization results of DT-DSATMO under six cross-domain scenarios. (**a**) Scenario S1, (**b**) Scenario S2, (**c**) Scenario S3, (**d**) Scenario S4, (**e**) Scenario S5, and (**f**) Scenario S6.

Figure 16 presents the damage localization results of DT-DSATMO across six cross-domain sensing scenarios. In each subplot, solid circles “●” indicate the true damage locations, stars “★” denote the predicted positions, and arrows visualize the localization errors. The different colors of the symbols in each scenario represent different damage locations. It can be observed that as the number of labeled physical space sensor-feature samples increases, the predicted points progressively converge toward the true locations, the arrows shorten, and the predictions become more concentrated and consistent.

**Figure 17:**
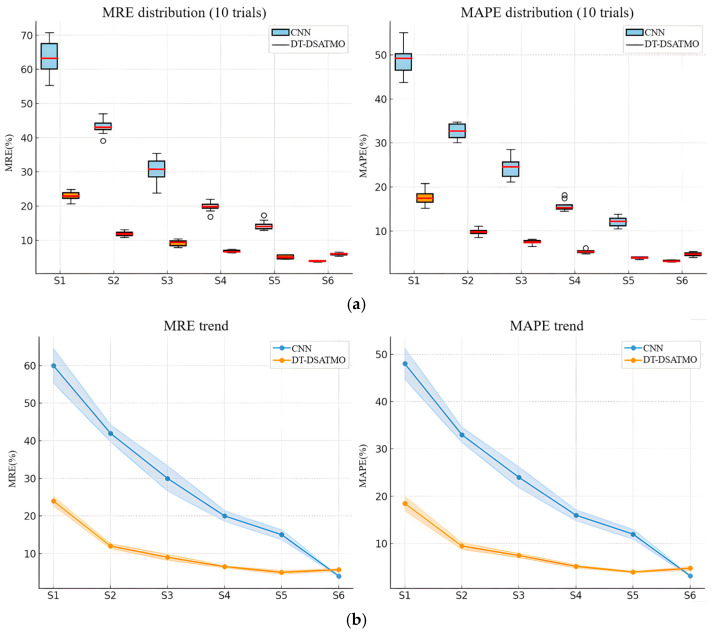
Analysis of the proposed DT-DSATMO method: (**a**) feature distribution comparison; (**b**) trend evaluation.

Figure 17 presents a comprehensive comparison of the localization errors for the two methods across six cross-domain sensing scenarios (S1–S6). The first method corresponds to a conventional CNN, while the second method is the proposed DT-DSATMO. Table 4 summarizes the commonly used evaluation metrics, where lower mean absolute percentage error (MAPE) and mean relative error (MRE) values indicate better performance [38]. The upper panels show box plots for the MAPE and MRE across ten independent trials, and the lower panels present the mean values, with error bars representing one standard deviation. In the small-sample cross-domain sensing scenarios S1–S5, DT-DSATMO achieved lower mean errors and reduced variance, demonstrating superior cross-domain generalization and robust training. In the S6 scenario with abundant experimental sensor-feature samples, DT-DSATMO maintained performance comparable to the fully supervised CNN. These results validate the two-stage adversarial transfer learning digital twin framework as an effective approach for Lamb wave-based damage localization with limited sensing data and domain-shift conditions.

**Figure 18:**
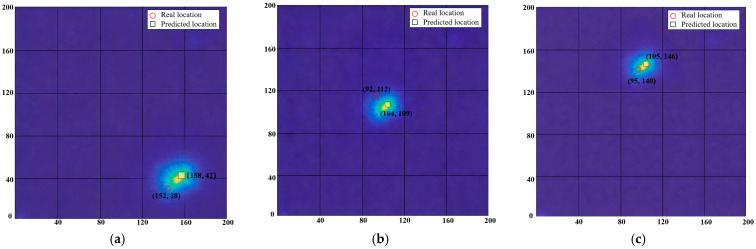
Imaging results of DAS for damage at different locations. (**a**) D1, (**b**) D2, and (**c**) D3.

**Figure 19:**
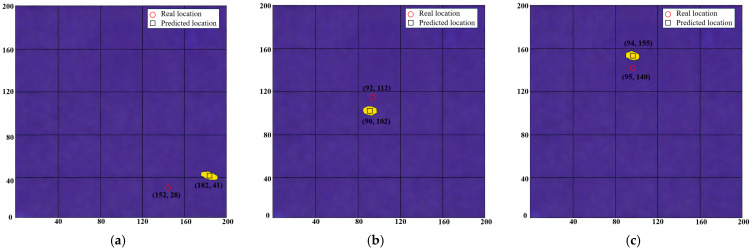
Imaging results of EPI for damage at different locations. (**a**) D1, (**b**) D2, and (**c**) D3.

In addition to the comparison with the deep learning-based CNN model, this study further verifies the superiority of the proposed DT-DSATMO method against two traditional physics-driven methods (DAS and EPI). Their damage imaging results at different positions are shown in Figure 18 (DAS) and Figure 19 (EPI), respectively, and the localization errors are summarized in Table 5. In Figure 18 and Figure 19, red circles represent the real damage locations, while black squares indicate the predicted damage locations. As shown in Figure 18, the DAS method features simple calculation and strong real-time performance for rapid damage estimation, but lacks accuracy for unknown positions, as it only relies on time-delay alignment and signal superposition, making it vulnerable to noise and distortion. Figure 19 shows that the EPI method has a systematic bias toward the sensor array due to its heavy dependence on sensor-damage distance and wave propagation characteristics. The deviations in propagation time measurement cause offsets in elliptical overlapping areas, leading to sensor-proximal imaging. Moreover, both methods are limited by sensor quantity and propagation path selection. More paths may improve accuracy but increase data acquisition and calculation complexity, sacrificing real-time performance. Comprehensive analysis shows that while DAS and EPI meet basic damage localization needs, their overall performance is significantly inferior to the DT-DSATMO method.

Overall, the proposed method expands and aligns the semantic distribution of the simulated sensing domain via the domain-adaptive conditional generator (DACG) and captures domain-invariant yet discriminative temporal features through domain adversarial adaptation (DAA). Multi-objective optimization dynamically balances localization accuracy, adversarial consistency, and higher-order alignment.

### 4.3. Ablation Analysis on Stage-Wise Contributions

To validate the effectiveness of the two-stage framework in DT-DSATMO, an ablation study was conducted using the S2 cross-domain sensing scenario. Four model configurations were designed based on whether the first stage, DACG, and the second stage, DAA, were enabled, as summarized in Table 6.

**Figure 20:**
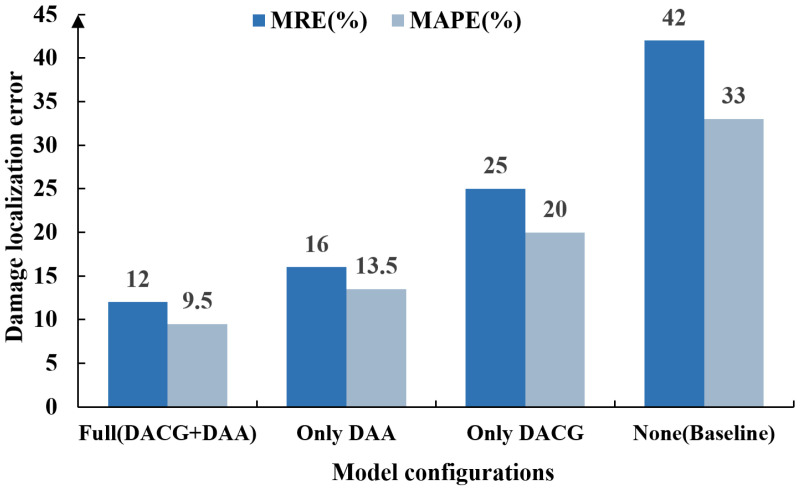
The localization error results for each model configuration.

Figure 20 shows the localization errors for each configuration. The complete DT-DSATMO achieves the best performance, outperforming all other variants. In contrast, the baseline model without any feature augmentation or domain adaptation exhibits the highest errors, highlighting the severe impact of the distribution shift between digital-domain and physical-domain sensing signals. These results confirm that both the DACG and DAA stages are essential for robust cross-domain generalization in small-sample Lamb wave-based damage localization.

The only DAA configuration retained the framework’s second stage, yielding a substantial error reduction relative to the baseline model. The MRE decreased from 42% to 16% and the MAPE decreased from 33% to 13.5%. These improvements demonstrate that DAA effectively mitigates the feature discrepancies between simulated and experimental sensor data. However, without the DACG stage, the model remains constrained by limited scenario diversity in the adversarial training set, indicating that adversarial adaptation alone cannot fully overcome the challenges posed by scarce sensor data. The Only DACG configuration employs conditional adversarial feature augmentation to enrich digital-domain sensing features. Although this achieves a moderate error reduction, the absence of adversarial alignment with the experimental data results in distribution mismatches, which in turn constrain cross-domain generalization.

The ablation results confirm that DACG improves feature coverage under small-sample conditions, while DAA enforces consistency across sensing domains. Their combined effect yields an approximate 71% reduction in both MAPE and MRE, resulting in enhanced cross-domain robustness and more accurate localization.

### 4.4. Model Generalization Under Extreme Small-Sample and Different Damage Sizes

**Figure 21:**
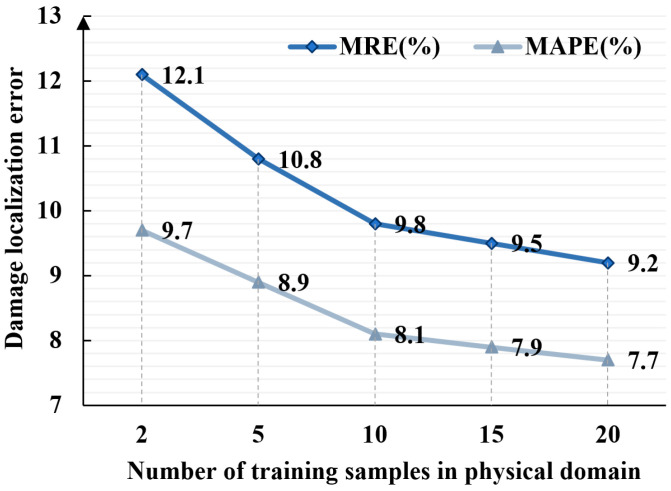
Variation in MAPE and MRE with the number of physical domain samples.

To further assess the cross-domain generalization capability of the proposed DT-DSATMO method under extreme small-sample conditions, experimental scenarios were designed in which the number of training samples in the physical domain progressively reduced. The number of digital domain samples was maintained at 400, while the number of physical domain training samples was set to 20, 15, 10, 5, and 2 for the tasks denoted as T-20, T-15, T-10, T-5, and T-2, respectively. Each task was independently repeated ten times using the same network architecture and optimization hyperparameters, and the results were averaged to reduce the effect of random variation. The corresponding experimental results are shown in Figure 21.

As shown in Figure 21, when the number of experimental domain training samples decreases from 20 to 10, the DT-DSATMO model maintains stable prediction performance, with the MAPE below 8.1% and the MRE below 9.8%. This indicates that the multi-objective adversarial transfer mechanism effectively extracts domain-invariant features and aligns digital-domain and physical-domain sensing signals under limited supervision. When only five experimental domain samples are available, the MAPE increases to 8.9% and the MRE rises to 10.8%, with both feature alignment and regression accuracy experiencing a moderate decline.

When only two experimental samples are available, the MAPE stands at 9.7% and the MRE at 12.1%, slightly exceeding the MAPE and MRE values observed without experimental data. This degradation stems from alignment bias caused by insufficient data, which may lead to the domain discriminator overfitting local patterns, thereby undermining the consistency of global features. Nevertheless, the increase in error remains moderate, indicating that the model retains robustness under severe data scarcity.

**Figure 22:**
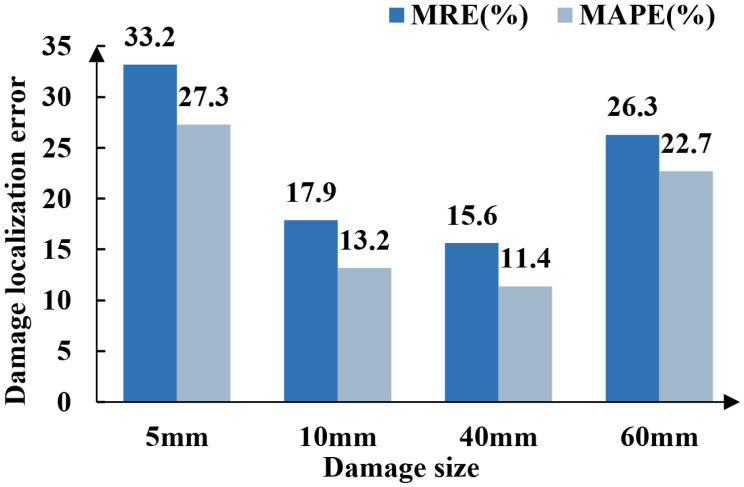
Localization errors for different unseen damage sizes.

These results demonstrate that, despite limited sensing data availability, DT-DSATMO exhibits strong adaptability. To further evaluate the algorithm’s sensitivity to defect size and its generalization capability, additional experiments use artificial damage with diameters of 5 mm, 10 mm, 40 mm, and 60 mm, beyond the 20 mm samples used for training. These cases are processed using the same data preprocessing and inference pipeline without retraining. As shown in the newly added Figure 22, the model maintains strong diagnostic performance across these unseen damage sizes, demonstrating robustness and effective generalization to a wider range of structural damage scenarios. DACG broadens the diversity of features in the digital domain, while DAA aligns cross-domain features to enhance generalization. Through multi-objective optimization, the framework jointly regulates localization accuracy, adversarial alignment, and higher-order constraints to avoid overfitting.

## 5. Conclusions

To resolve the conflict between the reliance on experimental sensor data and cross-domain location performance in Lamb wave-based digital twin structural health monitoring, this study proposes a two-stage adversarial transfer method for digital twin-driven damage localization using multi-objective optimization. The main conclusions are as follows:

1. Digital-twin modeling: A physics-guided simulation-sensing data augmentation framework is introduced to enhance the quality of digital-domain features. This approach embeds guided-wave propagation knowledge into a hierarchical augmentation strategy, generating damage-sensitive features that maintain physical interpretability.
2. Cross-domain localization: A two-stage adversarial transfer framework is introduced to bridge simulated and experimental perception domains, enabling reliable damage localization. The first stage enhances simulated features, while the second stage improves cross-domain consistency through a shared encoder adversarial network. A multi-objective optimization strategy balances accuracy and alignment, ensuring robust localization with scarce sensing data.
3. Case results: DT-DSATMO achieved reductions of approximately 71% in MAPE and MRE across diverse damage scenarios. Despite minor alignment-induced degradation observed in only two experimental sensor samples, the model maintained strong cross-domain performance and practical applicability.

In summary, the proposed framework provides a physically interpretable and lightweight solution for cross-domain Lamb wave damage localization, demonstrating robust performance with limited sensor-acquired data and cross-domain conditions. It offers a practical approach for digital-twin-based structural health monitoring when physical domain sensing signals are scarce. Future work will extend the model to broader structural configurations, sensor layouts, and environmental conditions.

## Figures and Tables

**Table 1:** Material property parameters of CFRP structure.

Parameters		Values	Units
Elastic modulus	E1	135	Gpa
	E2	8.8	Gpa
	E3	8.8	Gpa
Shear modulus	G12	4.7	Gpa
	G13	4.7	Gpa
	G23	3	Gpa
Poisson’s ratio	v12	0.3	-
	v13	0.3	-
	v23	0.3	-
Density		1570	kg/m^3^
Layers		16	-
Layering directions		[0°/90°/0°/90°/0°/90°/0°/90°]s	-

**Table 2:** Mechanical and electrical properties of the PZT and adhesive used in the model.

Type	Parameters		Values	Units
PZT	Elastic modulus	E1	71	Gpa
		E2	71	Gpa
		E3	62	Gpa
	Shear modulus	G12	20	Gpa
		G13	20	Gpa
		G23	21	Gpa
	Poisson’s ratio	v12	0.4	-
		v13	0.4	-
		v23	0.35	-
	Piezoelectric strain constants	d31	−160	pc/N
		d32	−160	pc/N
		d33	362	pc/N
		d15	561	pc/N
	Dielectric permittivity	ε11	1650	-
		ε33	1600	-
	Density		6720	kg/m^3^
Adhesive	Young’s modulus		2.6	Gpa
	Poisson’s ratio		0.3	-
	Density		1100	kg/m^3^

**Table 3:** Damage diagnosis tasks with different numbers of training samples across domains.

Scenario	Digital Domain Training Samples	Physical Domain Training Samples
S1	75	0
S2	400	0
S3	400	25
S4	400	50
S5	400	75
S6	400	100

**Table 4:** Damage localization error quantitative measures.

Quantitative Damage Indicators	Equation
Mean absolute percentage error (MAPE)	$MAPE=\frac{100\%}{n}\sum_{i=1}^{n}\left|\frac{{\hat{y}}_{i}-y_{i}}{y_{i}}\right|$
Mean relative error (MRE)	$MRE=\frac{1}{n}\sum_{n}^{i=1}\left[\sqrt{{\left(x_{T}^{i}-x_{P}^{i}\right)}^{2}+{\left(y_{T}^{i}-y_{P}^{i}\right)}^{2}/L}\right]\times 100\%$

**Table 5:** Damage location prediction results and localization error of DAS and EPI.

Traditional Method	Damage Sample	Real Location	Predicted Location	MRE (%)	MAPE (%)
DAS	D1	(152, 28)	(158, 42)	58	52
	D2	(92, 112)	(104, 109)		
	D3	(95, 140)	(105, 146)		
EPI	D1	(152, 28)	(182, 41)	50	45
	D2	(92, 112)	(90, 102)		
	D3	(95, 140)	(94, 155)		

**Table 6:** Four model configurations for ablation analysis.

Model Configuration	Includes DACG	Includes DAA
Full (DACG + DAA)	Yes	Yes
Only DAA	No	Yes
Only DACG	Yes	No
None (Baseline)	No	No

## Data Availability

The data used to support the findings of this study are available from the corresponding author upon request.

## References

[1] Tu J., Yan J., Ji X., Liu Q., Qing X. (2024). Damage Severity Assessment of Multi-Layer Complex Structures Based on a Damage Information Extraction Method with Ladder Feature Mining. Sensors.

[2] Liu H., Huang M., Zhang Q., Liu Q., Wang Y., Qing X. (2024). Ultrasonic guided wave damage localization method for composite fan blades based on damage-scattered wave difference. Smart Mater. Struct..

[3] Mei L.-F., Yan W.-J., Yuen K.-V., Beer M. (2025). Streaming variational inference-empowered Bayesian nonparametric clustering for online structural damage detection with transmissibility function. Mech. Syst. Signal Process..

[4] Zhang Y., Wu X., Guo Q., Zhang D., Li C., Li D., Liu Y., Zhang J., Zhang P., Yan Y. (2025). Advances in sensors technologies for composites structural health monitoring. Compos. Struct..

[5] Qing X.P., Beard S.J., Kumar A., Ooi T.K., Chang F.-K. (2007). Built-in Sensor Network for Structural Health Monitoring of Composite Structure. J. Intell. Mater. Syst. Struct..

[6] Liu H., Liu Q., Zhang Q., Huang M., Wang Y., Qing X. (2026). Crest sequence compensation method for improving the accuracy of ultrasonic guided wave based damage detection under variable temperatures. Measurement.

[7] Balasubramaniam K., Sikdar S., Ziaja D., Jurek M., Soman R., Malinowski P. (2023). A global-local damage localization and quantification approach in composite structures using ultrasonic guided waves and active infrared thermography. Smart Mater. Struct..

[8] Bosse S., Lehmhus D., Kumar S. (2024). Automated Porosity Characterization for Aluminum Die Casting Materials Using X-ray Radiography, Synthetic X-ray Data Augmentation by Simulation, and Machine Learning. Sensors.

[9] Puder A., Zink M., Seidel L., Sax E. (2024). Hybrid Anomaly Detection in Time Series by Combining Kalman Filters and Machine Learning Models. Sensors.

[10] Sapidis G.M., Naoum M.C., Papadopoulos N.A. (2025). Electromechanical Impedance-Based Compressive Load-Induced Damage Identification of Fiber-Reinforced Concrete. Infrastructures.

[11] Huang X., Han M., Deng Y. (2024). A Hybrid GAN-Inception Deep Learning Approach for Enhanced Coordinate-Based Acoustic Emission Source Localization. Appl. Sci..

[12] Nie Q., Geng J., Liu C. (2026). A Review of Fault Diagnosis Methods: From Traditional Machine Learning to Large Language Model Fusion Paradigm. Sensors.

[13] Lai X., Yang L., He X., Pang Y., Song X., Sun W. (2023). Digital twin-based structural health monitoring by combining measurement and computational data: An aircraft wing example. J. Manuf. Syst..

[14] Sun L., Sun H., Zhang W., Li Y. (2024). Hybrid monitoring methodology: A model-data integrated digital twin framework for structural health monitoring and full-field virtual sensing. Adv. Eng. Inform..

[15] Aliakbari M., Mahmoudi M., Vadasz P., Arzani A. (2022). Predicting high-fidelity multiphysics data from low-fidelity fluid flow and transport solvers using physics-informed neural networks. Int. J. Heat Fluid Flow.

[16] Ritto T.G., Rochinha F.A. (2021). Digital twin, physics-based model, and machine learning applied to damage detection in structures. Mech. Syst. Signal Process..

[17] Huang Y., Qing X., Xie M. (2025). Balanced fidelity digital twin for structural damage monitoring based on Lamb wave multilevel feature enhancement and adaptive space interaction. Struct. Health Monit..

[18] Nerlikar V., Miorelli R., Recoquillay A., d’Almeida O. (2024). A physics-embedded deep-learning framework for efficient multi-fidelity modeling applied to guided wave based structural health monitoring. Ultrasonics.

[19] Absi G.N., Mahadevan S. (2016). Multi-fidelity approach to dynamics model calibration. Mech. Syst. Signal Process..

[20] Liao Y., Wang Y., Fang C., Yang X., Zeng X., Chronopoulos D., Qing X. (2025). Baseline-free damage detection and localization on composite structures with unsupervised Kolmogorov-Arnold autoencoder and guided waves. Mech. Syst. Signal Process..

[21] Ahmadzadeh M., Zahrai S.M., Bitaraf M. (2025). An integrated deep neural network model combining 1D CNN and LSTM for structural health monitoring utilizing multisensor time-series data. Struct. Health Monit..

[22] Das T., Guchhait S. (2025). A hybrid GRU and LSTM-based deep learning approach for multiclass structural damage identification using dynamic acceleration data. Eng. Fail. Anal..

[23] Zhao B., Qing X., Wang Y., Liu Q., Yan J., Wang Y., Liao Y. (2025). Impact monitoring based on domain adversarial transfer learning strategies. Smart Mater. Struct..

[24] Soleimani-Babakamali M.H., Soleimani-Babakamali R., Nasrollahzadeh K., Avci O., Kiranyaz S., Taciroglu E. (2023). Zero-shot transfer learning for structural health monitoring using generative adversarial networks and spectral mapping. Mech. Syst. Signal Process..

[25] Liu C., Chen Y., Xu X. (2025). Structural digital Twin for damage detection of CFRP composites using meta transfer Learning-based approach. Expert Syst. Appl..

[26] Sheng H.Y., Ye J.Q. (2002). A semi-analytical finite element for laminated composite plates. Compos. Struct..

[27] Tannhäuser K., Serrao P.H., Kozinov S. (2023). Second-Order Collocation-Based Mixed FEM for Flexoelectric Solids. Solids.

[28] Ozgun O., Kuzuoglu M., Mittra R. (2024). Self-Tuning Locally Conformal PML Mesh Truncation for 3-D Vector Finite Element Method. IEEE Trans. Antennas Propag..

[29] Ni Q., Ji J.C., Feng K., Halkon B. (2022). A fault information-guided variational mode decomposition (FIVMD) method for rolling element bearings diagnosis. Mech. Syst. Signal Process..

[30] Rubio-González C., del Pilar de Urquijo-Ventura M., Rodríguez-González J.A. (2023). Damage progression monitoring using self-sensing capability and acoustic emission on glass fiber/epoxy composites and damage classification through principal component analysis. Compos. Part B Eng..

[31] Li R., Li S., Xu K., Li X., Lu J., Zeng M., Li M., Du J. (2022). Adversarial domain adaptation of asymmetric mapping with CORAL alignment for intelligent fault diagnosis. Meas. Sci. Technol..

[32] Zeng H., Ren B., Zhang L., Zhang H., Cui L., Guo J. (2026). Enhanced small-sample aviation accident prediction via an improved WCGAN incorporating neuralprophet and gradient penalty. Reliab. Eng. Syst. Saf..

[33] Huang Y., Kang J., Liu L., Zhong X., Lin J., Xie S., Meng C., Zeng Y., Shah N., Brandon N. (2022). A hierarchical coupled optimization approach for dynamic simulation of building thermal environment and integrated planning of energy systems with supply and demand synergy. Energy Convers. Manag..

[34] Shan S., Qiu J., Zhang C., Ji H., Cheng L. (2016). Multi-damage localization on large complex structures through an extended delay-and-sum based method. Struct. Health Monit..

[35] Tian J., Liu M., Xiao H., Zhang Z., Yang W., Long Z., Leung C.M. (2023). Optimized ultrasonic total focusing imaging of diverse and multiple defects in crossply CFRP: Floquet wave theory, numerical simulation, and experimental validation. Mech. Syst. Signal Process..

[36] Leckey C.A.C., Wheeler K.R., Hafiychuk V.N., Hafiychuk H., Timuçin D.A. (2018). Simulation of guided-wave ultrasound propagation in composite laminates: Benchmark comparisons of numerical codes and experiment. Ultrasonics.

[37] Asadzadeh M., Zouraris G.E. (2024). On the convergence of a linearly implicit finite element method for the nonlinear Schrödinger equation. Stud. Appl. Math..

[38] Sun B., Wu Z., Feng Q., Wang Z., Ren Y., Yang D., Xia Q. (2023). Small Sample Reliability Assessment With Online Time-Series Data Based on a Worm Wasserstein Generative Adversarial Network Learning Method. IEEE Trans. Ind. Inform..

